# Preliminary Study to Assess the Impact of Dietary Rutin on Growth, Antioxidant Capacity, and Intestinal Health of Yellow Catfish, *Pelteobagrus fulvidraco*

**DOI:** 10.3390/ani13213386

**Published:** 2023-10-31

**Authors:** Apeng Liu, Xing Lu, Zhehui Ji, Lixue Dong, Jiayuan Jiang, Juan Tian, Hua Wen, Zhen Xu, Guohuan Xu, Ming Jiang

**Affiliations:** 1 Department of Aquatic Animal Medicine, College of Fisheries, Huazhong Agricultural University, Wuhan 430070, China; 2 Yangtze River Fisheries Research Institute, Chinese Academy of Fishery Sciences, Wuhan 430223, China; 3 Shenzhen Aohua Group Co., Ltd., Shenzhen 518109, China; 4 State Key Laboratory of Freshwater Ecology and Biotechnology, Institute of Hydrobiology, Chinese Academy of Sciences, Wuhan 430072, China; 5 State Key Laboratory of Applied Microbiology Southern China, Guangdong Institute of Microbiology, Guangdong Academy of Sciences, Guangzhou 510070, China

**Keywords:** *Pelteobagrus fulvidraco*, growth performance, antioxidant capacity, tissue morphology, intestinal flora

## Abstract

**Simple Summary:**

Rutin is a collection of natural compounds that possess a distinctive polyphenolic structure of flavonoids. The present preliminary study aimed to investigate the impact of dietary rutin supplementation on the growth performance, antioxidant capacity, and intestinal health of yellow catfish. Three diets supplemented with different levels of rutin (0, 100 mg/kg and 500 mg/kg rutin) were fed to juvenile tilapia for 56 days. Results revealed that supplementing with 100 mg/kg rutin enhanced growth, antioxidant capacity, and intestinal health in yellow catfish. However, the administration of rutin at a dosage of 500 mg/kg did not yield any additional advantages but potentially exhibited adverse effects on yellow catfish. This study has demonstrated the potential of rutin as a novel feed additive in aquafeed.

**Abstract:**

This research aimed to examine the effects of dietary rutin supplementation on growth, body composition, serum biochemical indexes, liver enzyme activities and antioxidant-related genes expression, intestinal morphology, and microbiota composition of juvenile yellow catfish (*Pelteobagrus fulvidraco*). Rutin was added to the basal diets at doses of 0 (control), 100 mg/kg, and 500 mg/kg. Each diet was fed randomly into three tanks, each tank containing 30 fish with an initial body mass of (10.27 ± 0.62) g. The feeding trial was conducted in an indoor recirculating aquiculture system at 28 °C for 56 days. According to the findings, the inclusion of 100 mg/kg rutin significantly improved the growth performance of yellow catfish and reduced the feed conversion ratio; however, the growth promotion effect was diminished when the diet was supplemented with 500 mg/kg of rutin. The inclusion of 500 mg/kg rutin in the diet significantly reduced the level of crude lipid and protein of the whole fish. Serum activities of alkaline phosphatase, albumin, and total protein were all significantly increased when fish were fed the diet supplemented with 500 mg/kg rutin, while serum glucose was significantly lower compared to the control group. Meanwhile, dietary rutin at a concentration of 500 mg/kg significantly induced the hepatic mRNA expressions of antioxidant-related genes (including *Cu/Zn-SOD*, *Mn-SOD*, *CAT*, *GPx*) and inflammatory-associated genes (including *TNFα*, *IL-10*, *LYZ*). Incorporating rutin at doses of 100 mg/kg and 500 mg/kg into the diets resulted in a notable increase in superoxide dismutase (SOD) activity, while simultaneously reducing malondiadehyde (MDA) content in the liver and intestine. Intestinal villus height, villus width, muscular thickness, and lumen diameter were significantly increased with the administration of 500 mg/kg of dietary rutin. Gut microbial diversity analysis indicated that supplementing diets with 100 mg/kg and 500 mg/kg rutin significantly enhanced the abundance of *Cetobacterium* while decreasing *Plesiomonas* richness. In conclusion, dietary rutin levels at 100 mg/kg could enhance the growth, antioxidant capability, and intestinal health of yellow catfish under present experimental conditions.

## 1. Introduction

Rutin is a collection of natural compounds that possess a distinctive polyphenolic structure of flavonoids [1]. It exhibits an abundance of phenolic hydroxyl groups, particularly catechol functional groups, which facilitate easy absorption in the small intestine or binding with endogenous proteins within the intestinal tract. Consequently, it could reach intestinal tissue and be transported to other tissues via the bloodstream. Rutin has demonstrated various biological activities including anti-inflammatory effects [2], antioxidant properties [3], antiviral capabilities [4], as well as vasoactive and neuroprotective functions [5]. These remarkable attributes hold potential for applications aimed at reducing oxidative stress, alleviating inflammation, promoting growth, and enhancing immunity in organisms. In addition, its safety, low toxicity, and lack of biological residues make it a promising candidate for the development of functional feed additives in aquaculture. For example, supplementation with 0.8 and 1.0 g/kg of rutin in the diet can improve the growth performance and muscle quality of grass carp (*Ctenopharyngodon idella*) [6]. The administration of 1.5 g/kg of rutin in the diet of silver catfish (*Rhamdia quelen*) resulted in increased antioxidant enzyme activities in the brain, liver, kidney, and gills while also promoting growth [7]. Furthermore, dietary supplementation with rutin can improve the liver antioxidant response of Nile tilapia and reduce liver and muscle fiber damage induced by T-2 toxin, thereby improving the health status of Nile tilapia (*Oreochromis niloticus*) [8].

Yellow catfish (*Pelteobagrus fulvidraco*) is a widely cultured fish species in China. Its production reached about 0.58 million tons, which ranked tenth in total freshwater fish output [9]. Currently, fish cultivation predominantly relies on high-density ponds. However, this intensive culture pattern inevitably leads to metabolic disorders in fish and deterioration of water quality. With the increasing market demands and expansion of large-scale farming, yellow catfish have been found to exhibit a range of issues including growth retardation, compromised antioxidant capacity, and weakened disease resistance [10]. Consequently, ensuring the health and enhancing the growth performance of yellow catfish remains a significant concern.

Therefore, we hypothesized that rutin could serve as a functional additive in the yellow catfish diet to enhance their growth performance and health status. To test this hypothesis, we conducted a feeding experiment employing a single-factor gradient test to investigate the impact of dietary rutin on the growth performance, body composition, serum biochemical indexes, liver enzyme activities, antioxidant-related genes, intestinal morphology, and microbiome composition of yellow catfish. The obtained findings could provide valuable insights into comprehending the overall effects of dietary rutin on yellow catfish. This gathered information establishes a solid foundation for the practical utilization of rutin in feed manufacturing processes.

## 2. Materials and Methods

### 2.1. Ethics Statement

Yellow catfish are commonly farmed fish and are not endangered. The welfare of experimental fish involved in this study was approved by the Animal care and use committee of the Yangtze River Fisheries Research Institute, Chinese Academy of Fisheries Sciences. The approval code is YFI2022JM03.

### 2.2. Experimental Diets

The test rutin used in the experiment was ultra violet pure, and its purity was >97% (Shanghai yuanye Bio-Technology Co., Ltd., Shanghai, China). Based on the previous literature [11,12], dietary rutin supplementation levels were set at 0, 100 mg/kg, and 500 mg/kg, named R0, R100, and R500, respectively. Table 1 displayed the formulation and proximate compositions of the experimental diets. The experimental diet production procedures have been described in detail in a published paper from the same laboratory [13]. To briefly introduce the process, all the dry components were pulverized and sifted through a 60-mesh sieve, then measured based on the formulation (Table 1). After 10 min of mixing, the dry ingredients were slowly added with fish oil and soybean oil to continue stirring until there were no obvious oily particles, and 45% distilled water were added to continue stirring for 5 min. The strips were extruded by a small meat grinder through a 1 mm sieve and dried by a dryer. The strips were simply broken by a crusher and then passed through a 20-mesh screen. The small pellets were preserved in a refrigerator set at −20 °C for future use.

### 2.3. Experimental Fish and Feeding Management

The experimental yellow catfish were provided by a local fish breeding facility. The experimental fish were acclimated for 4 weeks in an indoor recirculating aquiculture system (RAS) at Yangtze River Fisheries Research Institute, Chinese Academy of Fishery Sciences. During the domestication period, the fish were hand-fed three times a day with apparent satiation. Prior to the official feeding trial, the fish were fasted for 24 h. Afterwards, 270 yellow catfish with initial weight (10.27 ± 0.62 g, *n* = 30) were chosen and randomly divided into 9 tanks (R = 0.82 m, water depth = 0.75 m). Three tanks were randomly divided into groups and fed one of the test diets. The fish were fed three times a day (8:30, 12:30 and 16:30), and the feeding rate was 3–5% of body weight. The fish were weighed once every two weeks, and the feeding rate was adjusted according to the change of body weight. The feeding trial went on for 56 days. Feed intake and mortality of test fish were recorded daily. The filter sand tank of the RAS was backwashed with about 10% fresh water before feeding every morning and afternoon. The water temperature was kept at 28 °C. During the culture experiment, water quality parameters were measured once a week. The main water quality parameters were dissolved oxygen > 5 mg/L, pH: 6.5–7.5, total ammonia < 0.2 mg/L, nitrite < 0.05 mg/L.

### 2.4. Sample Collection and Processing

At the end of the feeding trial, the fish were fasted for 24 h, then counted and weighed in buckets, and the weight gain rate and specific growth rate were calculated. Six fish per tank were anesthetized with 75 mg/L MS-222(Sigma, St. Louis, MO, USA), and their body length and weight were measured. Blood was drawn through the tail artery and allowed to stand for 4 h, then centrifuged at 960× *g* at 4 °C for 10 min to isolate the supernatant. The viscera and liver were dissected on ice and weighed to calculate the viscerosomatic index (VSI), hepatosomatic index (HSI), and condition factor (CF). Part of the liver and midgut of 3 fish per tank were placed in frozen tubes, frozen in liquid nitrogen, and stored in the refrigerator at −80 °C for the determination of liver metabolomics, antioxidant indexes, and intestinal microorganisms. An additional portion of the liver and the midgut of 6 fish per tank was used for the preparation of HE tissues sections for histological observation.

### 2.5. Proximate Analysis

Approximate analyses of whole fish and experimental diets were performed according to the method of the Association of Official Agricultural Chemists (AOAC, 2000 [14]). In brief, crude protein was determined by the Kjeldahl method; crude lipid content was determined by Soxhlet ether extraction. Moisture was determined by freeze-drying. Ash content was checked for 24 h at 550 °C using a muffle furnace. The gross energy was determined using an isothermal automatic calorimeter.

### 2.6. Serum Biochemical Parameters Detection

The contents of serum total protein (TP) (Sysmex, 290618), albumin (ALB) (Sysmex, 290615), triglyceride (TG) (Sysmex, 80945, 80946), total cholesterol (TCHO) (Sysmex, 290723, 290724), glucose (GLU) (Sysmex, 290713, 290714), and the activities of alkaline phosphatase (ALP) (Sysmex, 290701, 290702), aspartate aminotransferase (AST) (Sysmex, 290705, 290706) and alanine aminotransferase (ALT) (Sysmex, 290703, 290704) were detected by an automatic biochemical analyzer (Chemistry-800, Kobe, Japan). Diagnostic reagents were obtained from Sysmex Wuxi Co., Ltd. (Wuxi, China) according to standard protocols.

### 2.7. Analysis of SOD and MDA in Liver and Intestine

The tissue was thoroughly homogenized with 10 volumes (*w*/*v*) of ice-cold saline solution; it was centrifuged at 960× *g* for 10 min at 4 °C to separate the supernatant. Superoxide dismutase (SOD, A001-3-1) and malondialdehyde (MDA, A003-1-1) were measured by biochemical kits (Nanjing Jiancheng Bioengineering Institute, Nanjing, China).

### 2.8. Histopathology Evaluation

The fixed liver and intestinal samples were trimmed, dehydrated, embedded in paraffin, sectioned with a thickness of 5 μm, stained with HE, and sealed by dehydration. Images were examined using a light microscope (Leica DM2500, Leica, Solms, Germany). Five fields were randomly selected from each intestinal section to measure the number, height, width, diameter of mucosal fold, and muscular layer thickness using Image pro plus 6.0 software (IPP 6.0, Media Cybernetics Inc., Bethesda, MD, USA).

### 2.9. Gut Microbiota Analysis

The midgut samples from the R0, R100, and R500 groups were collected for DNA extraction. After the measurement of DNA integrity and purity, about 2 ng/μL DNA for each sample was used for 16S rRNA sequencing. The primer sets including 338F (5′-ACTCCTACGGGAGGCAGCA-3′)/806R (5′-GGACTACHVGGGTWTCTAAT-3′) were utilized to amplify variable V3–V4 regions of the 16s rRNA. The sequencing was accomplished by the staff of Shanghai Meiji Biotechnology Co., Ltd. (Shanghai, China) with the Illumina Miseq platform. Bioinformatics analysis was described in detail following our recently published literature [15]. Representative sequences were assigned to operational taxonomic units (OTUs) with 97% similarity, and alpha diversity analysis and taxonomic composition analysis at the phylum or genus level were performed based on the clustering results.

### 2.10. Quantitative Real-Time PCR (qRT-PCR)

Total RNA was extracted from liver samples of groups R0, R100, and R500 using Trizol reagent (Takara Biotechnology, Tokyo, Japan), according to the manufacturer’s instructions. RNA integrity was verified by 2% agarose gel electrophoresis, and the concentration and purity were determined using a Nanodrop spectrophotometer (Thermo Fisher Scientific, Waltham, MA, USA). Two μg of RNA from each sample was reverse transcribed to first-strand cDNA using the primer-scriptTM RT kit (Takara Biotechnology).

qRT-PCR was performed using an ABI 7500 Real-Time PCR system with a reaction volume of 20 µL, 10 µL SYBR Premix Ex Taq II (Takara Biotechnology), 8.2 µL double-distilled water, 1 µL cDNA template, and 0.4µL primer (10 µM) included. All reactions were performed three times. Superoxide dismutase 1 (*Cu/Zn-SOD*), superoxide dismutase 2 (*Mn-SOD*), catalase (*CAT*), glutaredoxin *3* (*GPx*), alpha-induced protein 8-like 1 (*TNFα*), *IL-10* (interleukin 10), lysozyme g-like (*LYZ*), and ribosomal protein L13a (*Rpl13a*) were selected as target genes for detection. These primer sequences are listed in Table 2. The reference gene *β-actin* was used for gene normalization. Relative mRNA expression levels were calculated according to the 2^−ΔΔCt^ method [16].

### 2.11. Statistical Analysis

SPSS20.0 (IBM Corp., Armonk, NY, USA) was used for one-way analysis of variance (one-way ANOVA) of the experimental data, and Duncan’s multiple comparison analysis was used to analyze the significance of the difference between groups. All data results were expressed as mean ± standard deviation (mean ± SD, *n* = 3), and *p* < 0.05 indicated significant difference.

## 3. Results

### 3.1. Growth Performance

Table 3 presents the growth performance of yellow catfish fed different test diets. The fish fed diets R100 and R500 had a significantly lower feed conversion ratio (*p* < 0.05) than the fish fed diet R0. The fish fed with diet R100 exhibited a significantly higher weight gain rate and specific growth rate compared to the fish fed with diet R0 (*p* < 0.05), and there was no significant difference between group R500 and group R100 (*p* > 0.05). There were no significant differences on the hepatosomatic index, condition factor, and the viscerosomatic index of yellow catfish among the groups (*p* > 0.05).

### 3.2. Whole-Body Proximate Compositions

The proximate compositions of whole fish are presented in Table 4. The crude protein content of whole fish in the control group was significantly higher than that in the R100 and R500 groups (*p* < 0.05). The crude lipid content of the whole fish in the R500 group was significantly lower than that of the other two groups (*p* < 0.05). Dietary rutin supplementation did not affect the moisture and ash contents of the whole body (*p* > 0.05).

### 3.3. Serum Biochemistry Parameters

Table 5 displays serum biochemical parameters of the test fish. The contents of total protein (TP) and albumin (ALB) as well as alkaline phosphatase (ALP) activity in serum were significantly elevated in the R500 group compared to the R0 group (*p* < 0.05). Fish in the R100 and R500 groups had notably reduced levels of glucose (GLU) and activities of alanine transaminase (ALT) compared to the fish in the control group (*p* < 0.05). Nonetheless, the experimental groups did not display any noteworthy variations in serum total protein (TP), triglyceride (TG), total cholesterol (TCHO), and aspartate aminotransferase (AST) activity (*p* > 0.05).

### 3.4. SOD Activity and MDA Content in Liver and Intestine

The superoxide dismutase (SOD) activity and malondiadehyde (MDA) content of yellow catfish are displayed in Table 6. Dietary rutin led to a significant increase in SOD activity in both liver and intestine (*p* < 0.05), and the MDA content was significantly lower in the R100 and R500 groups compared to the R0 group (*p* < 0.05).

### 3.5. Gene Expressions Analysis in Liver

**Figure 1:**
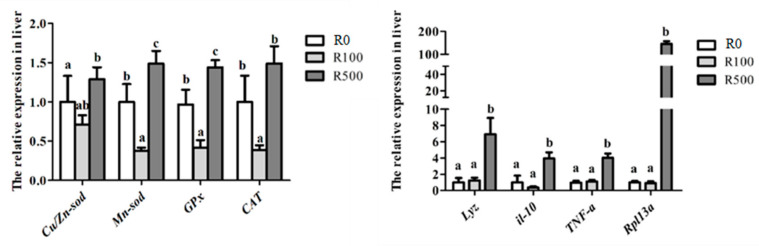
Relative mRNA expressions of antioxidant-related genes (including *Cu/Zn-SOD*, *Mn-SOD*, *CAT* and *GPx*) and inflammatory-associated genes (including *TNFα*, *IL-10*, *LYZ* and *Rpl13a*) in the liver of yellow catfish fed three diets with rutin supplementation at 0 (R0), 100 mg/kg (R100), and 500 mg/kg (R500), respectively. Results are presented as the means ± SD (*n* = 3), Values in the same line sharing different superscript letters are significantly different at *p* < 0.05.

The results of genes expression analysis are shown in Figure 1. The transcriptional expressions of hepatic antioxidant-related genes (including *Cu/Zn-SOD*, *Mn-SOD*, *CAT*, and *GPx*) and inflammation-related genes (*TNFα*, *IL-10*, *LYZ*, *Rpl13a*) in the R500 group were significantly up-regulated among the three groups. The R100 group had the lowest transcriptional expression of *Mn-SOD*, *CAT*, and *GPx* among the three groups. However, there was no significant difference in the expression of inflammation-related genes between the R100 group and the control group.

### 3.6. Histopathology Evaluation in Liver and Intestine

**Figure 2:**
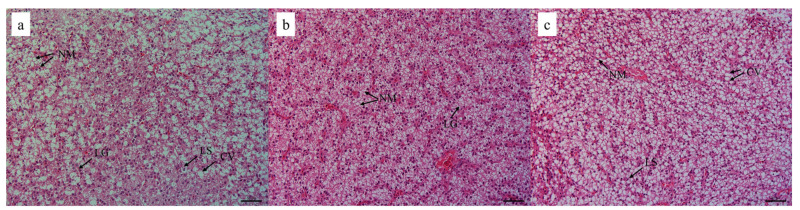
Histologically observed liver of yellow catfish fed with rutin supplementation at (**a**) 0, (**b**) 100 mg/kg, and (**c**) 500 mg/kg, respectively (×400, scale bar = 20 μm). NM, nuclear migration; CV, cellular vacuolization; LS, hepatocyte swelling; LG, intercellular gap.

**Figure 3:**
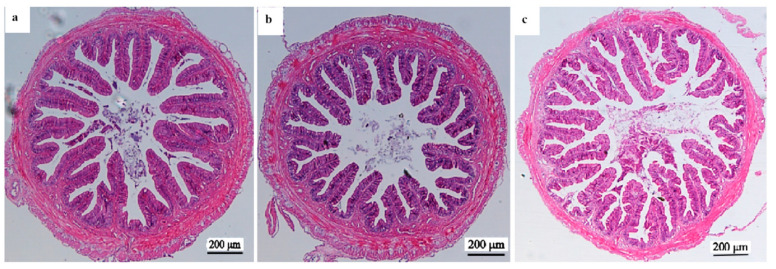
Histologically observed midgut of yellow catfish fed three diets with rutin supplementation at 0 (**a**), 100 mg/kg (**b**), and 500 mg/kg (**c**), respectively (100×, scale bar = 200 μm).

Figure 2 shows the liver histology of yellow catfish. The hepatocytes in fish fed a 100 mg/kg rutin diet were intact and tightly arranged, while fish in the control group or the 500 mg/kg rutin supplementation group exhibited larger cell volume, serious nuclear migration, hepatocyte vacuolation, and augmented intercellular space. Figure 3 and Table 7 list the intestinal morphological changes. The highest value of intestinal villus length was detected in fish fed a diet with 100 mg/kg rutin. Fish in the R0 or R100 group had higher values of muscular thickness and lumen diameter than fish in the R500 group (*p* < 0.05).

### 3.7. Gut Microbial Diversity Analysis

As shown in Figure 3, the average number of operational taxonomic units (OTUs) in the R0 group, R100 group, and R500 group were 54, 121, and 420, respectively. The total number of OTUs was 31 among all dietary treatments. There were 3, 36, and 317 unique OTUs in each group, respectively.

**Figure 4:**
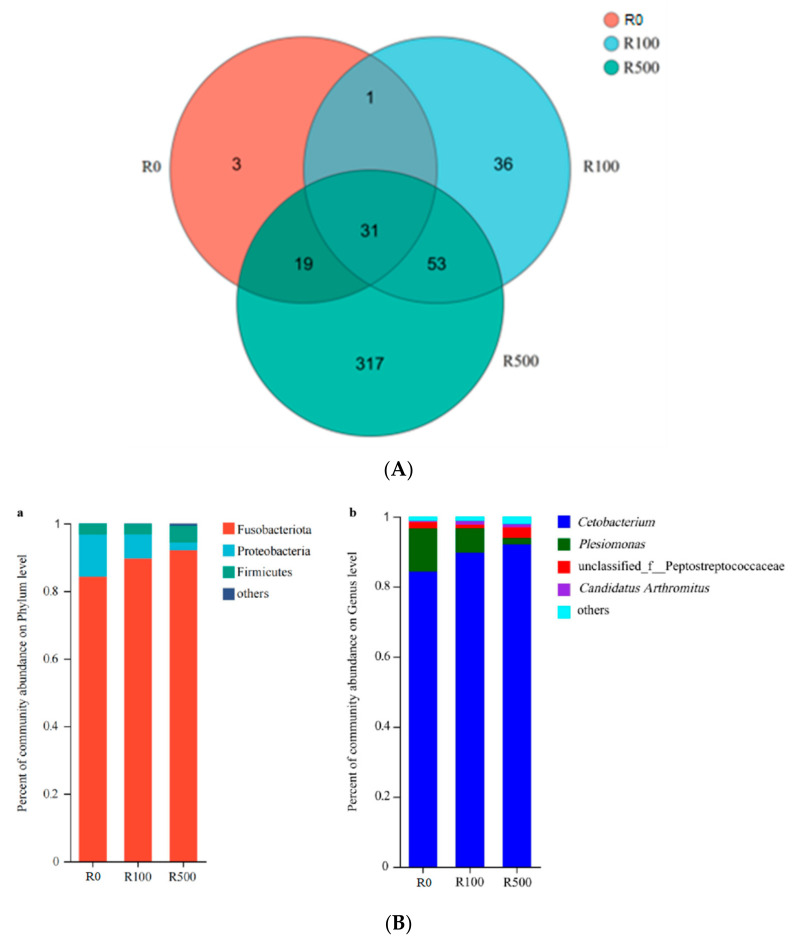
(**A**) Venn diagram showing the distribution of operational taxonomic units (OTUs) shared by yellow catfish fed three diets with rutin supplementation at 0 (R0), 100 mg/kg (R100), and 500 mg/kg (R500), respectively. (**B**) Relative abundance of gut flora at the phylum (**a**) and genus (**b**) level in yellow catfish fed three diets with rutin supplementation at 0 (R0), 100 mg/kg (R100), and 500 mg/kg (R500), respectively.

Table 8 shows the abundance and diversity of intestinal bacteria in three experimental groups. There was no significant difference in the Shannon and Simpson index among the three groups (*p* > 0.05). The Chao and Ace index in the R500 group was significantly higher than that in R0 group (*p* < 0.05). At the phylum level, Fusobacteria, Proteobacteria, and Firmicutes were the dominant phylum in the gut flora of yellow catfish (Figure 4A). Compared with the R0 group, the abundance of Fusobacteria and Firmicutes increased while Proteobacteria decreased in both the R100 and R500 groups. At the genus level, *Cetobacterium*, *Plesiomonas*, *Peptostreptococcaceae*, and *Candidatus Arthromitus* were the dominant genera in the gut flora of yellow catfish (Figure 4B). The richness of *Cetobacterium* increased while *Plesiomonas* decreased in both the R100 and R500 groups compared with the R0 group.

## 4. Discussion

In this study, the inclusion of 100 mg/kg rutin significantly enhanced growth performance and reduced the feed conversion ratio in yellow catfish. However, supplementation with 500 mg/kg rutin did not exhibit a significant effect on growth. Previous studies have demonstrated that flavonoid extracts and plant-derived compounds can act as growth promoters in aquaculture species by modulating antioxidant enzyme activities, immune responses, intestinal morphology, and microbial compositions [17,18]. In the case of yellow catfish, the observed improvement in growth performance and feed utilization may be attributed to enhanced antioxidant capacity and improved gut health. These factors effectively reduce oxidative stress and facilitate nutrient absorption within the fish gut. Similar findings have also been reported for other commercially important fish species such as Nile tilapia [8] and grass carp [6]. However, a relatively higher concentration of rutin in the diet may contain certain anti-nutritional substances, leading to a decrease in feed intake and subsequently causing growth retardation in fish. Moreover, supplementation of dietary rutin at 100 mg/kg significantly reduced the crude protein content in the whole body, indicating that rutin could potentially function as a regulatory factor involved in nutrient metabolism within this particular species.

SOD is an important indicator for the assessment of oxidative damage in fish. SOD has a function in scavenging the free radicals to protect the body from oxidative stress, and its activity is positively associated with the health conditions in fish species [19]. Rutin is reported to trigger the increment in SOD activity, remove excess reactive oxygen species (ROS), and therefore, improve the antioxidant system [20]. Likewise, the addition of rutin in the diet significantly increased the activities of many kinds of antioxidant enzymes in the brain, liver, kidney, and gill of silver catfish [7]. In the current experiment, the upward trend or significant elevation of SOD activity in both liver and intestine was detected when fish were fed the diets with 100 mg/kg, demonstrating that rutin has ameliorative roles on the antioxidant capacity of yellow catfish, and relieves oxidative-caused injury by counteracting the generation of free radicals. This data may further evidence that rutin could serve as a potent agent in the improvement of antioxidative response in aquatic organisms [21]. MDA is one of several end products of the lipid peroxidation chain and indirectly reflects the degree of oxidative damage [22]. In the present study, MDA in the liver and intestine of yellow catfish was found to be inversely correlated with increasing dietary rutin concentrations, which may indicate that rutin is able to prevent peroxidation levels in fish tissues. Similar results have been found in rats [23].

Moreover, the mRNA expressions of antioxidant-related genes were examined in the liver. Cu/Zn-SOD is the most studied of superoxide dismutase; Cu is responsible for the catalytic activity and Zn is mainly in charge of the maintenance of its structure [24]. Mn-SOD is located in the soluble matrix of the mitochondria and has crucial roles in eliminating ROS in cells [25]. The product encoded by the *CAT* gene is a major antioxidant enzyme that converts hydrogen peroxide to oxygen and water in an energy efficient way [26]. GPx represents a key enzyme in the cellular machinery that catalyzes the reduction of hydroperoxides [27]. The present data showed that dietary rutin at 500 mg/kg significantly induced the transcriptional expression of *Cu/Zn-SOD*, *Mn-SOD*, *CAT*, and *GPx* genes, which may further support the enhancement of the antioxidant response in response to dietary rutin treatment. Furthermore, several genes, including *TNFα*, *IL-10*, *LYZ* in liver, were significantly up-regulated as fish were fed the diet with 500 mg/kg rutin, indicating that rutin could exert an anti-inflammatory effect on yellow catfish. A similar pattern of response has been reported on tilapia [28]. Fish fed the 100 mg/kg rutin diet had the lowest transcriptional expression of Mn-SOD, CAT, and GPx genes, suggesting that appropriate rutin supplementation had protective effects against oxidative stimulation.

Serum biochemical parameters generally reflect the fish physiological and metabolic conditions under nutritional manipulation or environmental stress [29]. TP could indicate protein metabolism in fish and maintain the balance of intravascular osmotic pressure [30]. ALB is considered a key modulator of plasma oncotic pressure within the vascular space [31]. ALP is an important non-specific immune enzyme for evaluating the fish immunity status in aquaculture [32]. In this study, dietary rutin at a concentration of 500 mg/kg significant elevated the values of serum TP, ALB, and ALP, indicating the beneficial effects of rutin on the enhancement in immune response and metabolic ability of yellow catfish. Previous findings on mammals also confirmed that dietary rutin could effectively increase the levels of serum TP and ALB, and delay the progression of liver fibrosis by down-regulating the expression of transforming growth factor-β1 (TGF-β1) and collagen I [33]. In addition, the significant reduction of serum GLU was observed in fish fed the rutin-supplemented diets in comparison with that of fish fed the control diet. This may be explained by the fact that flavonoid substances could stimulate the liver to liberate the glucose, induce the activation of insulin receptors or pathways, and thereby facilitate the absorption of glucose in serum [34]. This observation is in accordance with that of Nazer et al. [35], who indicated that dietary rutin can significantly decrease the blood glucose concentrations in rainbow trout (*Oncorhynchus mykiss*). In addition, the change in blood glucose is correlated with hepatic enzymes activities, which may suggest that rutin has a beneficial influence on liver function. Notably, reduced AST activity observed in fish fed with rutin further supports its potential for improving overall liver function when compared to the control diet.

H&E staining is commonly used for histopathology evaluation in fish due to that it can clearly illustrate cellular structures with remarkable signatures [36]. Excessive rutin intake has been implicated in impairing the health of liver tissue in the GIFT strain of tilapia [28]. In this research, the liver cellular structures were intact and tightly arranged when fish were fed the 100 mg/kg rutin diet. However, the liver cells treated with 500 mg/kg rutin in the diet showed larger cell volume, serious nuclear migration, hepatocyte vacuolation, and augmented intercellular space. It might be attributed to the occurrence of apoptotic cells or pro-inflammatory cytokines triggered by a high dose of rutin. The intestinal tract is the primary organ involved in fish digestion, nutritional absorption, and intestinal immunity. Some typical indicators such as villus numbers, villus length, muscular thickness and lumen diameter are used for the assessment of intestinal health [37]. Generally, the strong ability of nutrient absorption is a positive association with the greater absorptive area and increased intestinal villi [21]. Muscular thickness is closely related to the rhythmic contraction of the gut tract and efficient digestion of chyme [38]. In this study, the significant increase in values of villus length, muscular thickness, and lumen diameter were observed in fish fed the 100 mg/kg rutin diet, indicating that rutin has beneficial effects on intestinal morphological structures, and thus, effectively prompt its absorptive capability as well as nutrient consumption.

There exists a substantial population of microorganisms within the fish intestine which has established a dynamic and intricate microenvironment over an extended period of evolution [39]. The gut microbiota is known to uphold homeostasis and influence nutrient absorption, pathogen assimilation, as well as growth and development in fish [40]. Furthermore, the intestinal flora plays a pivotal role in regulating host immunity and maintaining immune homeostasis [41]. In the present study, Fusobacteria, Proteobacteria, and Firmicutes were identified as the predominant phyla within the intestinal flora of yellow catfish. These findings align with those reported by Wu et al. [42]. Notably, Proteobacteria represents the largest phylum encompassing bacteria or pathogens; an elevated abundance of Proteobacteria can disrupt the gut microecological environment in animal hosts while concurrently increasing disease risks [43]. Firmicutes have been demonstrated to possess the capacity for carbohydrate transformation, cellulose decomposition promotion, and polysaccharide fermentation. They also enhance fish digestion and nutrient absorption while aiding in the maintenance of gut immune system homeostasis in hosts [44]. In our study, the inclusion of rutin at levels of 100 mg/kg and 500 mg/kg in diets significantly augmented the abundance of Fusobacteria and Firmicutes, but reduced the richness of Proteobacteria. This suggests that appropriate dietary supplementation with rutin may potentially mitigate stress-related diseases. At the genus level, *Cetobacterium, Plesiomonas*, and *Peptostreptococcaceae* were identified as dominant genera within the gut microbiota of yellow catfish. *Cetobacterium* can produce vitamin B_12_ in the process of carbohydrates fermentation and play crucial functions in the nutritional metabolism [45]. Pseudomonas is a common pathogen existing in aquatic water and fish body, of which *Peptostreptococcaceae* predominated in gut diseases [46]. The present investigation showed that the increased abundance of *Cetobacterium* and the decreased *Plesiomonas* richness were determined on fish fed the diets with 100 mg/kg or 500 mg/kg rutin. These data suggested that dietary rutin has positive effects on the improvement of intestinal structure and microbial composition, which is beneficial to the gut health of yellow catfish.

## 5. Conclusions

In summary, the present results demonstrated that dietary supplementation of rutin at 100 mg/kg could improve the growth, antioxidant capability, and intestinal health of yellow catfish. However, the administration of rutin at a dosage of 500 mg/kg did not yield any additional advantages but potentially exhibited adverse effects on yellow catfish. The determination of the optimal quantity of rutin to be included in the diet of yellow catfish will be conducted in forthcoming investigations.

## Figures and Tables

**Table 1:** Formulation and proximate composition of the experimental diets (% air-dried matter).

Ingredients ^1^ (%)	R0	R100	R500
Fish meal	30	30	30
Soybean meal	20	20	20
Cottonseed meal	10	10	10
Corn gluten meal	10	10	10
High-gluten flour	17.20	17.19	17.15
Wheat gluten	2	2	2
Fish oil	3	3	3
Soybean oil	3	3	3
Ca(H_2_PO_4_)_2_	1.5	1.5	1.5
Vitamin premix ^2^	1	1	1
Mineral premix ^3^	2	2	2
Rutin	0	0.01	0.05
Vitamin C	0.1	0.1	0.1
Choline chloride	0.2	0.2	0.2
Total	100	100	100
Proximate composition (%)			
Moisture	5.68	6.13	5.86
Crude protein	43.14	43.56	43.31
Crude lipid	8.62	8.62	8.74
Ash	8.73	8.77	8.73
Gross Energy (KJ/g)	20.13	20.24	20.19

^1^ The ingredients in formula were purchased from the Tongwei Feed Company (Wuhan, China). ^2^ The vitamin premix provided the following per kg of diets: vitamin A 5000 IU, vitamin D_3_ 2000IU; vitamin E 60 mg; vitamin B_1_ 5 mg; vitamin B_2_ 20 mg; vitamin B_6_ 10 mg; vitamin C 120 mg; vitamin K_3_ 5 mg; inositol 400 mg; nicotinic acid 120 mg; calcium pantothenate 10 mg; folic acid 1 mg; biotin 0.1 mg. ^3^ The minerals premix provided the following per kg of diet: Ca(CH_3_CHOHCOO)_2_ 6540 mg; FeSO_4_ 42.5 mg; MgSO_4_ 1340 mg; NaH_2_PO_4_ 1744 mg; NaCl 870 mg; AlCl_3_ 3 mg; KIO_3_ 2.5 mg; KCl 1500 mg; CuCl_2_ 2 mg; MnSO_4_ 16 mg; CoCl_2_ 20 mg; ZnSO_4_ 60 mg.

**Table 2:** Primers used in this study.

Gene Name	Gene Bank NO.	Forward Sequences (5′-3′)	Reverse Sequences (5′-3′)	Tm (°C)
*Cu/Zn-SOD*	XM_027171881.2	GGCGGAGATGATGAAAGT	GAAAGGAAGCGGTGAAAC	60.1
*Mn-SOD*	XM_027166181.2	TGGTGCTTGCTATGGTGA	GGCTTGAATCCCTTGCTG	56.1
*Rpl13a*	XM_027160195.2	GCTGCGCTGGAGAGGCTGAAGGTGT	CGGTTCAATAAGGTTCTGCT	60.0
*CAT*	XM_027163801.2	TCTGTTCCCGTCCTTCATCC	ATATCCGTCAGGCAATCCAC	59.4
*GPx*	XM_027163146	ATCTACATTGGCTTGGAAAC	GAAAGTAGGGACTGAGGTGA	60.0
*TNFα*	XM_027160151.2	AACCGAAAGGAAGCACAGAA	TCACGGCAATCGTTTAGGAG	61.6
*IL-10*	XM027144360.1	CTCCTCCCCCTGAGGATTCA	CGGATCACGGCGTATGAAGA	59.3
*Lyz*	XM_047802086	GGAGCACATCAGACAAGGCA	CCTACTCCGGCATTGTAGGC	62.1
*β-actin*	XM_027148463.2	TTCGCTGGAGATGATGCT	CGTGCTCAATGGGGTACT	55.4

*Cu/Zn SOD*, superoxide dismutase 1; *Mn-SOD*, superoxide dismutase 2; *Rpl13a*, ribosomal protein L13a; *CAT*, catalase; *GPx*, glutare doxin 3; *TNF*α, alpha-induced protein 8-like 1; *IL-10*, interleukin 10; *Lyz*, lysozyme g-like.

**Table 3:** Growth performance and feed utilization of yellow catfish fed diets containing different rutin levels for 8 weeks.

Group ^1^	R0	R100	R500
IBW	10.75 ± 0.53	9.97 ± 0.21	10.10 ± 0.11
FBW	36.10 ± 1.63	41.19 ± 2.3	35.73 ± 1.18
WG	235.71 ± 2.02 ^a^	314.12 ± 28.09 ^b^	253.91 ± 12.73 ^ab^
SGR	2.16 ± 0.02 ^a^	2.53 ± 0.22 ^b^	2.25 ± 0.11 ^ab^
FCR	1.64 ± 0.10 ^b^	1.15 ± 0.13 ^a^	1.07 ± 0.12 ^a^
CF	1.73 ± 0.03	1.68 ± 0.02	1.70 ± 0.07
VSI	7.22 ± 0.28	6.43 ± 0.39	7.18 ± 0.51
HSI	1.51 ± 0.08	1.25 ± 0.01	1.40 ± 0.09
SR	93.98 ± 3.09	94.62 ± 2.16	96.32 ± 4.31

Data were presented as mean ± SD (*n* = 3) of three replicates per treatment. Values in the same line sharing different superscript letters are significantly different at *p* < 0.05. IBW (g/fish) = initial mean weight; FBW (g/fish) = final mean weight; WG (percent weight gain, %) = (FBW − IBW)/IBW × 100; SGR (specific growth rate, %/day) = 100 × (ln FBW—ln IBW)/feeding days; FCR = feed intake per tank/(total final fish weight − total initial fish weight + dead fish); CF (condition factor, g/cm^3^) = (body weight, g)/(body length, cm)^3^ × 100; VSI (viscerosomatic index, %) = 100 × (viscera weight, g)/(body weight, g); HSI (hepatosomatic index) = 100 × (g liver weight)/(g body weight); SR (survival rate) = 100 × (final fish number)/(initial fish number). ^1^ The diets R0, R100 and R500 contained with dietary rutin levels 0, 100, 500 mg/kg, respectively.

**Table 4:** Whole-body composition of yellow catfish fed diets containing different rutin levels for 8 weeks.

Group ^1^	R0	R100	R500
Moisture (%)	75.50 ± 0.62	77.00 ± 0.47	76.83 ± 0.17
Crude protein (%)	15.69 ± 0.1 ^b^	13.57 ± 0.3 ^a^	14.25 ± 0.29 ^a^
Crude lipid (%)	5.96 ± 0.23 ^b^	5.95 ± 0.19 ^b^	5.03 ± 0.16 ^a^
Ash (%)	5.12 ± 0.36	4.63 ± 0.21	4.78 ± 0.45

Data were presented as mean ± SD (*n* = 3) of three replicates per treatment. Values in the same line sharing different superscript letters are significantly different at *p* < 0.05. ^1^ The diets R0, R100, and R500 contained dietary rutin levels of 100, 500 mg/kg, respectively.

**Table 5:** Serum biochemical parameters of yellow catfish fed diets containing different rutin levels for 8 weeks.

Group ^1^	R0	R100	R500
ALB (g/L)	9.31 ± 0.87 ^a^	9.49 ± 0.91 ^a^	10.95 ± 0.18 ^b^
ALP (U/L)	33.83 ± 8.13 ^a^	41.50 ± 2.66 ^a^	55.00 ± 8.00 ^b^
ALT (U/L)	45.33 ± 1.52 ^b^	26.58 ± 3.16 ^a^	24.00 ± 2.00 ^a^
AST (U/L)	333.17 ± 38.81	331.33 ± 35.23	315.00 ± 19.47
TCHO (mmol/L)	4.10 ± 0.63	4.40 ± 0.42	4.80 ± 0.25
TG (mmol/L)	5.40 ± 1.49	5.01 ± 0.72	4.84 ± 0.89
TP (g/L)	33.9 ± 2.92 ^a^	32.91 ± 3.66 ^a^	38.21 ± 0.35 ^b^
GLU (mmol/L)	9.84 ± 2.22 ^b^	6.49 ± 2.02 ^a^	4.61 ± 0.22 ^a^

Data were presented as mean ± SD (*n* = 3) of three replicates per treatment. Values in the same line sharing different superscript letters are significantly different at *p* < 0.05. TP, total protein; ALB, albumin; TG, triglyceride; TCHO, total cholesterol; GLU, glucose; ALP, alkaline phosphatase; AST, aspartate transaminase; ALT, alanine transaminase. ^1^ The diets R0, R100, and R500 contained dietary rutin levels of 0, 100, 500 mg/kg, respectively.

**Table 6:** SOD and MDA activities in both liver and intestine of yellow catfish fed diets containing different rutin levels for 8 weeks.

Group ^1^	R0	R100	R500
Liver			
SOD (U/mg prot)	135.04 ± 8.99 ^a^	209.24 ± 12.87 ^b^	244.92 ± 5.89 ^c^
MDA (nmol/mg prot)	1.31 ± 0.04 ^b^	1.04 ± 0.10 ^a^	1.09 ± 0.01 ^a^
Intestine			
SOD (U/mg prot)	150.38 ± 1.45 ^a^	191.54 ± 4.83 ^b^	198.06 ± 6.19 ^b^
MDA (nmol/mg prot)	58.22 ± 0.63 ^b^	49.96 ± 2.22 ^a^	47.10 ± 3.21 ^a^

Data were presented as mean ± SD (*n* = 3) of three replicates per treatment. Values in the same line sharing different superscript letters are significantly different at *p* < 0.05. SOD, superoxide dismutase; MDA, malondialdehyde. ^1^ The diets R0, R100, and R500 contained dietary rutin levels of 0, 100, 500 mg/kg, respectively.

**Table 7:** Quantitative image analysis in liver of yellow catfish fed diets containing different rutin levels for 8 weeks.

Group ^1^	R0	R100	R500
Villus numbers	21.00 ± 1.63	20.33 ± 1.25	23.67 ± 1.25
Villus length (μm)	405.01 ± 93.82 ^ab^	325.3 ± 64.81 ^a^	476.16 ± 92.28 ^b^
Villus width (μm)	94.7 ± 6.94	94.35 ± 9.86	93.63 ± 13.94
Muscular thickness (μm)	56.32 ± 15.3 ^a^	54.05 ± 7.93 ^a^	72.76 ± 13.65 ^b^
Lumen diameter (μm)	1336.56 ± 106.72 ^a^	1266.29 ± 43.9 ^a^	1635.09 ± 242.97 ^b^

Data were presented as mean ± SD (*n* = 3) of three replicates per treatment. Values in the same line sharing different superscript letters are significantly different at *p* < 0.05. ^1^ The diets R0, R100, and R500 contained dietary rutin levels of 0, 100, 500 mg/kg, respectively.

**Table 8:** The abundance and diversity index in midgut of yellow catfish fed diets containing different rutin levels for 8 weeks.

Group ^1^	R0	R100	R500
Shannon	0.50 ± 0.11	0.42 ± 0.16	0.40 ± 0.15
Chao	28.9 ± 10.73 ^a^	47.01 ± 11.21 ^a^	104.18 ± 37.25 ^b^
Ace	42.72 ± 25.96 ^a^	55.18 ± 30.34 ^ab^	108.41 ± 36.96 ^b^
Simpson	0.76 ± 0.07	0.82 ± 0.10	0.85 ± 0.07

Data were presented as mean ± SD (*n* = 3) of three replicates per treatment. Values in the same line sharing different superscript letters are significantly different at *p* < 0.05. ^1^ The diets R0, R100, and R500 contained dietary rutin levels of 0, 100, 500 mg/kg, respectively.

## Data Availability

All data are available in the article.
